# Hyoid Bone Tracking in a Videofluoroscopic Swallowing Study Using a Deep-Learning-Based Segmentation Network

**DOI:** 10.3390/diagnostics11071147

**Published:** 2021-06-23

**Authors:** Hyun-Il Kim, Yuna Kim, Bomin Kim, Dae Youp Shin, Seong Jae Lee, Sang-Il Choi

**Affiliations:** 1 Department of Computer Science and Engineering, Dankook University, Yongin 16890, Korea; 72191491@dankook.ac.kr (H.-I.K.); 72210291@dankook.ac.kr (B.K.); 2 Department of Rehabilitation Medicine, Dankook University Hospital, Cheonan 31116, Korea; kimyuna727@dkuh.co.kr (Y.K.); sindae90@dkuh.co.kr (D.Y.S.); 3 Department of Rehabilitation Medicine, College of Medicine, Dankook University, Cheonan 31116, Korea; 4 Department of Computer Engineering, Dankook University, Yongin 16890, Korea

**Keywords:** dysphagia, hyoid bone, videofluoroscopy, deep learning

## Abstract

Kinematic analysis of the hyoid bone in a videofluorosopic swallowing study (VFSS) is important for assessing dysphagia. However, calibrating the hyoid bone movement is time-consuming, and its reliability shows wide variation. Computer-assisted analysis has been studied to improve the efficiency and accuracy of hyoid bone identification and tracking, but its performance is limited. In this study, we aimed to design a robust network that can track hyoid bone movement automatically without human intervention. Using 69,389 frames from 197 VFSS files as the data set, a deep learning model for detection and trajectory prediction was constructed and trained by the BiFPN-U-Net(T) network. The present model showed improved performance when compared with the previous models: an area under the curve (AUC) of 0.998 for pixelwise accuracy, an accuracy of object detection of 99.5%, and a Dice similarity of 90.9%. The bounding box detection performance for the hyoid bone and reference objects was superior to that of other models, with a mean average precision of 95.9%. The estimation of the distance of hyoid bone movement also showed higher accuracy. The deep learning model proposed in this study could be used to detect and track the hyoid bone more efficiently and accurately in VFSS analysis.

## 1. Introduction

Dysphagia is a common and growing healthcare concern because it can occur with age-related changes as well as various diseases, such as stroke, Parkinson’s disease, neuromuscular disease, and head and neck cancer, and frequently leads to serious complications, such as nutritional deficiencies, aspiration pneumonia, and suffocation [1,2,3]. The videofluoroscopic swallowing study (VFSS) is the gold standard examination for evaluating dysphagia and is the most objective and frequently used method [4]. During VFSS, the patient repeatedly swallows food boluses of various viscosities mixed with contrast while the swallowing process is recorded by fluoroscopy and then analyzed by clinicians or speech pathologists [4,5,6,7]. This process can visualize the anatomical structures and their dynamic functions involved in the swallowing process with bolus movement [4,5,7].

The hyoid bone is a horseshoe-shaped bone located in the anterior midline of the neck and is known as an important structure that participates in swallowing, speech, respiration, mastication, and maintaining the patency of the airway [8]. Among the anatomical structures associated with the swallowing process, the hyoid bone is a relatively radio-opaque structure and is easy to detect in VFSS images. Movement of the hyoid bone caused by contraction of the tongue base and suprahyoid muscles starts as the bolus passes into the pharynx and represents the initiation of physiologic swallowing [9,10]. Its horizontal and vertical movement is closely related to airway closure and upper esophageal sphincter opening, and reduced movement of the hyoid bone is associated with increased aspiration and pharyngeal residue [11,12]. Paik et al. reported that the extent and pattern of hyoid movement varies according to the etiology of dysphagia in two-dimensional kinetic swallowing motion analysis and suggested its applicability in differentiating the mechanism of dysphagia and treatment for reversing the mechanism [10]. However, in a clinical setting, manual tracking and quantitative measurement of hyoid bone movement is a labor-intensive and time-consuming task [13]. Moreover, the hyoid bone usually has unclear margins and varies in shape for each person. Therefore, manual tracking of the hyoid bone is inevitably prone to human error due to fatigue and individual subjective judgment, and wide intrarater and interrater variation has been shown [14]. Automatic tracking models of hyoid motion have been used to reduce human error and workload, and computer-assisted methods for kinematic analysis of hyoid bone movement have been proposed in a few studies [13,15,16,17]. However, these semiautomatic methods still require human judgment and manual input from clinicians and have a limitation of low performance and efficiency for application in clinical settings [13,15,16,17].

As deep learning technology has been developed and used for fast and efficient analysis of medical images acquired by techniques such as X-ray, computed tomography (CT), and magnetic resonance imaging (MRI) [18,19,20], recent studies have tried to apply deep learning to automate VFSS analysis [21,22,23,24,25,26,27]. However, we found only two studies that proposed deep learning models to detect the hyoid bone or track its movement in VFSS images [21,27]. Zhang et al. proposed the single shot multibox detector (SSD) model that can detect the hyoid bone fully automatically, but it showed less than perfect accuracy (mAP of the SSD-500 model = 89.14%), and tracking the whole movement of the hyoid bone was not attempted [27]. Lee et al. proposed a convolutional-neural-network (CNN)-based online learning algorithm, which can track hyoid motion and predict it even when it passes through the mandible and is blurred, but it still requires manual demarcation of a hyoid bone of appropriate size by humans in the first frame [21].

In this study, we aimed to develop a new deep learning model that could detect the hyoid bone and track its movement accurately and fully automatically without any human intervention in the whole process. Manual demarcation was totally excluded, and the distance was measured automatically by the model. We expect the model to be used efficiently for a larger number of dysphagic patients and to quickly assess the mechanism and severity of dysphagia, helping to determine appropriate treatment plans and feedback [21,27]. This model is also expected to accelerate the development of an automatic VFSS reading program to be applied in clinical practice.

## 2. Materials and Methods

### 2.1. Data Set

A total of 207 video files were selected from the VFSS database of the Department of Rehabilitation Medicine, Dankook University Hospital. The video files were recorded from patients who suffered from symptoms related to dysphagia between December 2017 and October 2020. When selecting the VFSS files, maximum effort was made to ensure that variables such as gender, age, viscosity of diet, and severity of dysphagia were distributed evenly. Among the videos, ten were excluded, which are difficult for humans to distinguish the cervical spines or hyoid bones, and 197 files were included in the data set. Each VFSS video was recorded at a speed of 30 frames per second (fps), and a total of 69,389 frames were obtained.

Each image was annotated once per 10 video frames, and data labeling was set with the hyoid bone as a target to track, the cervical spine for axis correction of the hyoid bone, and a 24 mm coin for depth correction of the hyoid bone. A total of three physicians skilled in VFSS analysis participated in the annotation process. All three physicians were experienced in VFSS analysis and rehabilitation of patients with swallowing disorders. One of them had more than twenty years of experience, and the other two had two to three years. Since every frame in the video has a high correlation with other adjacent frames, we adopted a method of annotating the fewest frames from as many videos as possible. We obtained 6632 frames from 197 videos through the above process. Then, they were split into training, validation, and test data sets at a ratio of 7:1:2 on each video and used in a deep learning network. The study protocol was approved by the Institutional Review Board of Dankook University Hospital (IRB No. 2021-03-010).

### 2.2. VFSS Protocols

The VFSS recording followed the protocol described by Logemann [6] with minor modifications. The VFSS video images were acquired from the lateral projection by fluoroscopic equipment (Versa 100, Shimadzu, Japan). Fluoroscopic images were stored digitally at a speed of 30 fps (frames per second) while the patients swallowed boluses with various viscosities mixed with contrast medium in a seated position. Ingredients with different consistencies were swallowed in the following order: 3 mL of thick liquid (water-soluble barium sulfate diluted to 70%), rice porridge, coagulated yogurt, and thin liquid (water-soluble barium sulfate diluted to 35%). In addition, a coin with a diameter of 24 mm was positioned on the skin surface of the lateral neck so that it did not overlap with the shadows of the cervical spine.

### 2.3. Object Detection in VFSS Using Deep Learning

**Figure 1:**
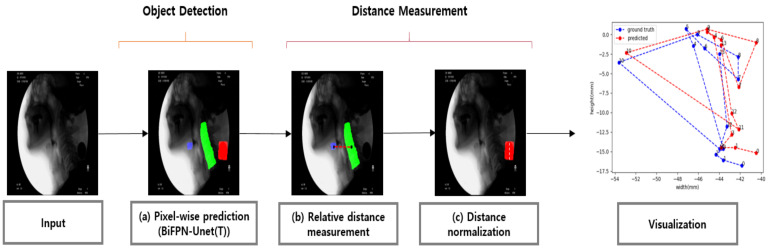
Schematic diagram of the proposed tracking system for the hyoid bone. Each frame of the video is used as an input in the deep learning network. (**a**) Each frame is classified by pixel through a deep learning segmentation model. (**b**) The relative distance between the hyoid bone and the cervical spine is calculated. (**c**) The relative distance obtained in (**b**) is converted to a normalized distance. Finally, the trajectory of the hyoid bone obtained through the above process is visualized.

In this section, we introduce the algorithm for object-of-interest detection in VFSS using a deep learning network for fully automatic hyoid bone tracking. The proposed method for tracking the hyoid bone is largely composed of the step of detecting the objects of interest (hyoid bone, cervical spine, and coin) in the VFSS image and the step of estimating the trajectory of the hyoid bone based on the cervical spine. Figure 1 shows the overall structure of the proposed method.

To detect the hyoid bone, cervical spine, and coin in fluoroscopic images, we used U-Net [28], which is widely used for the segmentation of biomedical images. The U-Net used in this paper adopted VGG16 [29] as the backbone. The backbone of the proposed network can be changed to other networks such as Resnet [30] and EfficientNet [31], depending on the user’s preference. U-Net is a fully convolutional network (FCN)-based model consisting of a convolutional encoder and a convolutional decoder that connects an encoder with a skip connection. The proposed U-Net takes a 224 × 224 image as input and extracts appropriate feature information to classify classes by pixel in the process of passing through 23 convolutional layers. U-Net can infer clearly in pixel units by concatenating the original information lost in the encoding process to the decoding process. However, U-Net has the disadvantage of combining feature information only in a top-down way that combines the feature information of deep layers with the feature information of shallow layers. Since weights are not used in the process of combining, feature information of different resolutions may contribute equally to the training process. Since the sizes of the hyoid bone, cervical spine, and coin are different in VFSS, we need a network that can effectively segment objects of various sizes. Therefore, we proposed a new network using a bidirectional feature pyramid network (BiFPN) [32] and bottleneck transformer (BOT) [33] to effectively segment various objects in VFSS (BiFPN-U-Net(T)).

**Figure 2:**
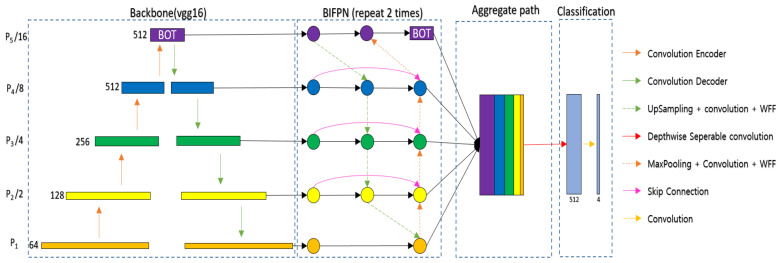
Schematic diagram of the proposed network (BiFPN-U-Net(T)).

Figure 2 shows the segmentation network with the BiFPN and BOT. The BiFPN uses a structure of both bottom-up and top-down approaches. The bottom-up approach, which combines feature information from P1 to P5, allows feature information with a large receptive field to consider details from the original information, and the top-down approach, which combines feature information from P5 to P1, allows feature information with a small receptive field to consider a wide range of contexts. However, simply adding different sizes of feature information can result in the same contribution in the process of combining feature information of different sizes. Therefore, in the BiFPN, different weights are multiplied and then added to make different contributions to creating new feature information in the process of combining feature information of different sizes. Additionally, the coin is usually attached to the patient’s neck during VFSS filming, and at this time, the hyoid bone can overlap with the mandible and disappear from the image, or the coin can go out of the field of view (FOV) of the filming device. To continuously track the location of an object even in the event of such occlusion, it is necessary to effectively utilize not only the information around the object but also the global context information. In general deep learning networks, the receptive field size is increased by stacking the convolution layer or increasing the size of the kernel [29]. As the receptive field widens, the model’s inference performance tends to increase because the network uses more information when inferring. However, when a deep learning network is designed by stacking convolution layers, practically, only a part of the receptive field is used for learning. The CNN uses a filter to extract feature information from images in a forward path, which tends to be referenced more when they are closer to the center than the outside. This can also affect the backpropagation process, as the feature information located at the outer edge has a smaller gradient. Therefore, the effective receptive field (the receptive field that directly affects the output value) tends to have the form of a Gaussian distribution [34]. This prevents deep learning networks from learning long-distance dependencies through the global context [35]. Therefore, the proposed method uses a BOT [33] on top of the BiFPN so that the network can reduce the overall computation and extract the global context effectively.

The BOT was proposed for applying global self attention to various computer vision tasks. It has been difficult to apply Transformer [36] to CNNs because the amount of computation increases quadratically according to the resolution of the feature map. The BOT uses a bottleneck structure to reduce the computation of the Transformer. To utilize the BOT effectively, we propose that the network applies the BOT only to P5, which has the smallest feature map size. We extract the global context by applying the BOT to P5, which is composed of three convolutional layers, as shown in Figure 2.

**Figure 3:**
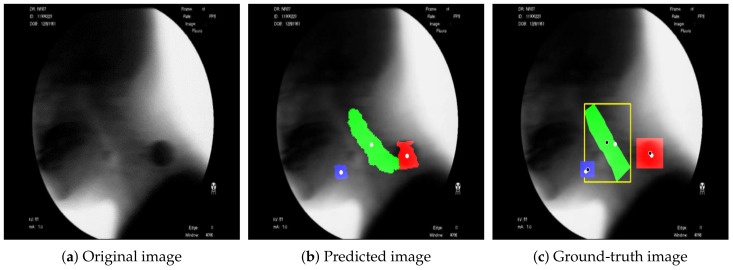
Segmentation results using the proposed method. (**a**) Original image; (**b**) segmentation results for the hyoid bone (blue), cervical spine (green), and coin (red). The average value of the coordinates of the detected pixels is indicated as a white spot. (**c**) Ground truth for the hyoid bone (blue), cervical spine (green: segmentation, yellow: detection), and coin (red). The average value of the coordinates of the detected pixels is indicated as a white spot, and the average value of the coordinates of the labeled pixels is indicated as a black spot.

Figure 3 shows the result of detecting the pixels corresponding to the hyoid bone, cervical spine, and coin using the proposed BiFPN-U-Net(T). In Figure 3b,c, the blue object denotes the hyoid bone, the green column denotes the cervical spine, and the red circle denotes the coin. The reference point for the position of the objects in analyzing the movement of the hyoid bone was defined as the average value of the coordinates of the detected pixels (white spots in Figure 3). As shown in Figure 3, the proposed method estimates the center points of the hyoid bone, cervical spine, and coin regions well.

### 2.4. Hyoid Bone Trajectory Prediction

The purpose of this study was to determine the swallowing function of the patient by observing the movement of the hyoid bone near the pharynx. Therefore, it was necessary to measure the relative movement of the hyoid bone based on the rigid structure from VFSS rather than the movement of the hyoid bone from the image coordinates. In general, head movement tends to increase when patients swallow boluses in VFSS, and sometimes a part of the head goes out of the image plane during the process of swallowing food. Therefore, the proposed method detects the cervical spine, which is relatively easy to detect due to the small variation in shape according to the individual, and calculates the relative position ($D_{x}^{R}$,$D_{y}^{R}$) of ($H_{x}$, $H_{y}$) to ($C_{x}$, $C_{y}$) from the center point ($C_{x}$, $C_{y}$) and the position of the hyoid bone ($H_{x}$, $H_{y}$) as follows [21,25]:

(1) $$\begin{array}{c} D_{x}^{R}=C_{x}-H_{x},\\ D_{y}^{R}=C_{y}-H_{y} \end{array}$$

where $D_{x}^{R}$ and $D_{y}^{R}$ are the distances in the horizontal and vertical directions between the center points of the hyoid bone and the cervical spine.

**Figure 4:**
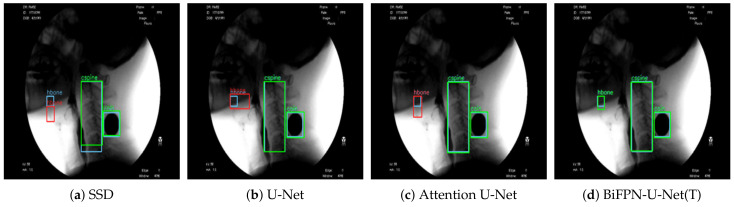
Detection results of each method when the hyoid bone overlaps with the mandible. The blue box denotes the ground truth, the green box denotes true positives, and the red box denotes false positives.

Since the scale of the subject in VFSS can vary depending on the resolution of the camera and the distance between the camera and the patient, it is necessary to normalize the movement in pixels to use the movement of the hyoid bone as an indicator of dysphagia. For this purpose, as shown in Figure 4, we normalized the scale of the image relative to the size of the coin by attaching a coin with a diameter of 24 mm around the neck during VFSS filming. Additionally, in the process of attaching the coin to the patient’s neck, if the camera axis and the surface of the coin are attached obliquely rather than vertically so that the coin looks like an oval (Figure 4), then the long axis of the ellipse is used as the approximate value of the diameter of the coin. In this experiment, the average diameter was 62.9 pixels, and the standard deviation was 7.6 pixels. As a result, we could confirm that there is a change in the VFSS scale according to the actual filming environment. Therefore, the proposed method defines the scale factor *s*, which refers to the size in the physical space occupied by a single pixel in the image, based on the actual diameter of the coin (24 mm) and the diameter of the coin in pixel units ($D_{pxls}$). Then, using *s*, the final relative position of the hyoid bone to the cervical spine ($N(D_{x}^{R}$), $N(D_{y}^{R}$)) was normalized in mm.

(2) $$\begin{array}{c} s=D_{pxls}/24 \end{array}$$

(3) $$\begin{array}{c} N\left(D_{x}^{R}\right)=s*D_{x}^{R},N\left(D_{y}^{R}\right)=s*D_{y}^{R} \end{array}$$

### 2.5. Network Training

For the experiment, an NVIDIA V100 32 GB GPU and Xeon Silver 4210 processor were used, and the libraries of Keras (v2.2.0) and TensorFlow (v.2.4.1) were used. The size of all images used in the experiment was fixed at 224×224. When applying a network used as a backbone to specific domains in deep learning studies, transfer learning [37] is used, which utilizes networks pretrained on large amounts of data, such as ImageNet [38]. However, unlike ImageNet, which consists of color images, VFSS data are grayscale. Therefore, to apply transfer learning from a network trained on color images, the color images used in the pretrained network have to be converted to black-and-white images [39]. In addition, in the case of VFSS images, the deviation of the distance between the object and the camera is not significantly large, whereas the images in the large-capacity database used for pretraining have various distances between the camera and the object, so the scale of the extracted feature information can also vary. It is not easy to obtain performance improvement through transfer learning. Therefore, transfer learning was not applied in this experiment, and we performed training by randomly initializing the weights of the backbone. The model was trained for 500 epochs, and if the loss did not drop for more than 20 epochs, the training was terminated by early stopping. In U-Net-based image segmentation, the number of pixels corresponding to the object being segmented (positive class) is relatively small compared to the other pixels (negative class). To alleviate the problem of unbalanced data between classes, we used focal loss [40]. Focal loss sets a small loss for the classes (here, the negative class) that are classified relatively often so that they are seldom updated. For the classes that are difficult to classify, focal loss makes them update with a large loss. This can make the model focus more on the difficult classes in training. The learning rate was set to 0.1 ∗ batch size/256 according to the batch size using the linear scaling learning rate method [41]. (Batch size = 8). The optimizer used Radam [42] for stable learning.

### 2.6. Performance Evaluation for Object Detection

We used the receiver operating characteristic (ROC) curve, which was mainly used in the detection problem to evaluate the detection performance for the hyoid bone, cervical spine, and coin in the proposed BiFPN-U-Net-based segmentation network. The segmentation network determines whether each pixel of an image is a positive or negative pixel. In our experiment, the pixels corresponding to the hyoid bone, cervical spine, and coin were defined as positive classes. The main metrics for performance evaluation are as follows: (1) accuracy: ratio of the correctly predicted observations to the total observations; (2) recall: the ratio of the correctly predicted positive observations to all observations in the actual class; (3) precision: the ratio of the correctly predicted positive observations to the total predicted positive observations; (4) Dice similarity (only for segmentation evaluation): the percentage of overlap between the predicted positive observations and all observations in the actual class.

### 2.7. Comparison with Other Models

To confirm the effectiveness of the proposed method (BiFPN-U-Net(T)), we performed a comparison experiment with previous models [21,27]. In the study of Zhang et al. [27], the SSD was used for robust operation on objects of various sizes by detecting each object from feature maps of various scales. In another study by Lee et al. [21], Attention U-Net was used with a featurewise gating mechanism to adjust the output values according to the importance of each feature in the upsampling process. For fair comparison, the VGG16 model was used as the backbone network of all models, and the training environments were the same.

## 3. Results

### 3.1. Performance in Object Detection

**Figure 5:**
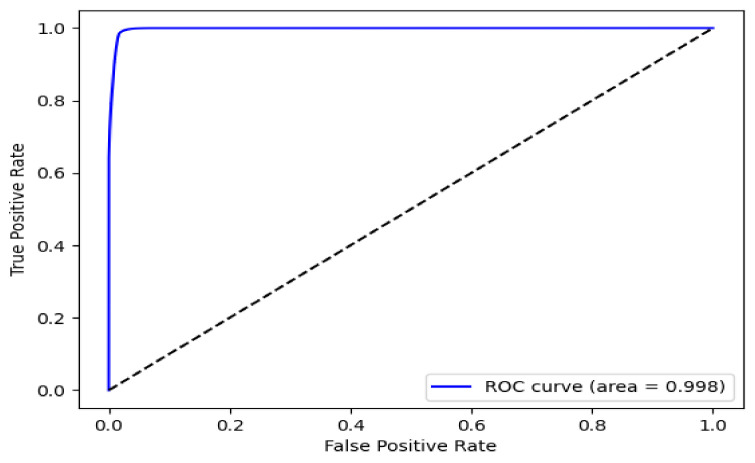
ROC curve for the pixelwise accuracy of BiFPN-U-Net(T).

Figure 5 shows the pixelwise accuracy of the proposed BiFPN-U-Net(T).

In Figure 5, the area under the curve (AUC) is 0.998, showing excellent performance. Table 1 shows the performance of each model based on the ground truth, as shown in Figure 3c. As shown in Table 1, the proposed BiFPN-based U-Net model showed 2.2%∼2.6% and 1.2%∼1.3% better performance in recall and Dice similarity, respectively, than other methods.

**Figure 6:**
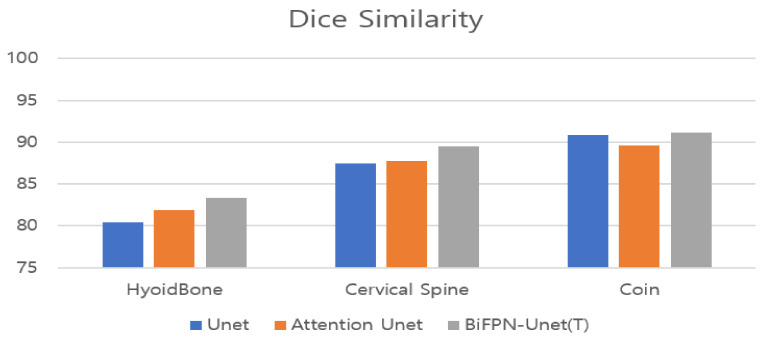
Performance of the proposed model in terms of Dice similarity per pixel.

The proposed method showed the best performance for each object among the hyoid bone, cervical spine, and coin (Figure 6). Since SSD is a detection network, not a segmentation network, the detection result of an object is presented in the form of a bounding box. For performance comparison with SSD, we defined the ground truth as the yellow line shown in Figure 3c and evaluated the performance of each model. The results of measuring the average precision for each object based on an intersection over union (IOU) of 0.5 are shown in Table 2.

**Figure 7:**
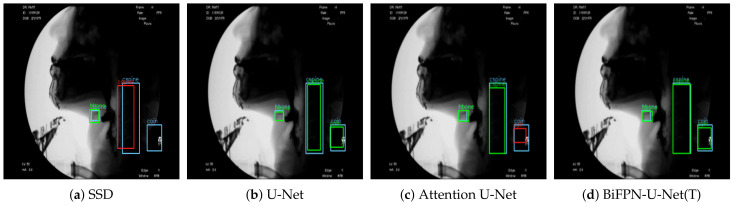
Detection results of each method when the coin is out of the field of view. The blue box denotes the ground truth, the green box denotes true positives, and the red box denotes false positives.

BiFPN-U-Net(T) shows the highest mean average precision (mAP) for the three types of objects, and the other methods differ in detection performance according to the type of object. Unlike the other methods, which have a large variation in detection performance depending on the type of object, BiFPN-U-Net(T) showed a high average precision of over 91% for all hyoid bones, cervical spines, and coins. Additionally, when the patient swallows the bolus, a part of the hyoid bone that often rises above the mandible in VFSS may be obscured (red box in Figure 4). In these circumstances, U-Net and Attention U-Net frequently cannot detect the hyoid bone, whereas a BiFPN-based network considering multiscale detection can detect the hyoid bone even when it is obscured, as shown in Figure 4. In addition, the coin attached to the neck often deviates from the field of view (FOV) due to the movement of the patient’s head during VFSS recording (Figure 7). Even in these circumstances, the proposed methods detected the coin more robustly than other methods.

### 3.2. Performance in Hyoid Bone Tracking

**Figure 8:**
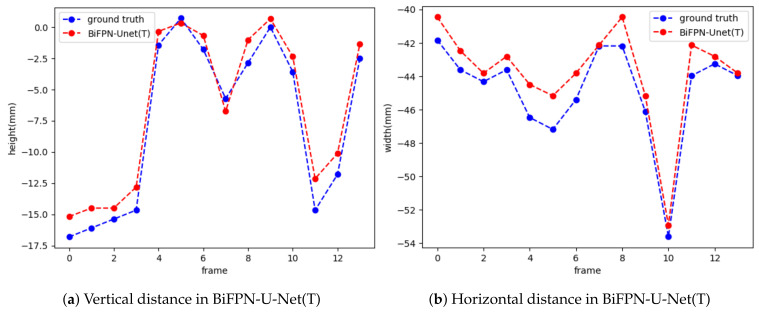
Visualization of distance measurements by hyoid bone tracking for a video.

To track the movement of the hyoid bone, we used the normalized distances ($N(D_{x}^{R})$, $N(D_{y}^{R})$), which are normalized from the center point of the cervical spine to the hyoid bone in the horizontal and vertical directions, as suggested in Section 2.4. Then, we measured the diagonal distance $D_{\left(C,H\right)}=\sqrt{N{\left(D_{x}^{R}\right)}^{2}+N{\left(D_{y}^{R}\right)}^{2}}$ from the horizontal and vertical distances. Figure 8 shows $N(D_{x}^{R})$ and $N(D_{y}^{R})$ measured by the proposed method for each frame in the video. The proposed method accurately predicted the position of the hyoid bone when compared with the ground truth. We also measured the distance of hyoid bone movement from the original position at the first frame to the maximum elevation in the horizontal, vertical, and diagonal directions.

Table 3 shows the ground truth labeled by the physicians and the prediction results of the deep learning models. We measured the distances only for frames where detections of all three types of objects (hyoid bone, cervical spine, coin) were achieved by each method. In Table 3, the number in parentheses next to the name of each method is the number of frames used to calculate the mean of distances. The proposed model showed the closest results to the ground truth and less standard deviation compared to other models, which means that the model stably and correctly tracks the hyoid bone. In addition, the proposed model showed the smallest root-mean-square error (RMSE) between the actual distance and the distance predicted by the models, except for the SSD in the horizontal direction (Table 4).

## 4. Discussion

The swallowing process consists of oral, pharyngeal, and esophageal stages according to the location of the bolus [6,43]. Normal pharyngeal swallowing includes two important functions: (1) food passage (the food bolus is propelled through the pharynx and upper esophageal sphincter to the esophagus) and (2) airway protection (the larynx and trachea are insulated from the pharynx during food passage to prevent food from entering the airway) [44]. The hyoid bone is a U-shaped bone that is connected to the thyroid cartilage and temporal styloid by ligaments [45]. In pharyngeal swallowing, the hyoid bone and larynx are pulled upward and forward by contraction of the suprahyoid muscles and thyrohyoid muscle, migrating the larynx under the tongue base [44]. The superoanterior excursion of the hyoid bone prevents laryngeal aspiration and promotes opening of the upper esophageal sphincter [46,47]. Therefore, the range of movement of the hyoid bone in VFSS can be an important index for evaluation of the swallowing function. The movement of the hyoid bone shows wide variation among individuals and can be influenced by the volume of the bolus and disease conditions [48]. A reduced range of hyoid movement may contribute to penetration–aspiration risk and increase pharyngeal residues in dysphagia [11]. Because the extent and pattern of hyoid movement has been reported to be different from that of normal individuals as well as according to the etiology of dysphagia, its analysis could be useful in differentiating the mechanism of dysphagia [10]. Therefore, kinematic analysis of hyoid movement in VFSS is important. However, it is not easy even for experts in VFSS analysis to detect and track the hyoid bone in the rapid swallowing process because the shape and density of the hyoid bone differ from person to person, and its margin is usually unclear [49]. Because many clinicians struggle to calibrate the kinematics of hyoid movement, it shows wide intrarater and interrater variation [14].

This study is the first to detect and track the hyoid bone without human intervention. A deep learning model that can identify the hyoid bone in a fully automatic manner has been reported, but tracking of the hyoid bone was not attempted in that model [27]. Another model for tracking the trajectory of the hyoid bone has been reported, but it still requires manual demarcation in the first-frame image [21]. We designed a robust network that can detect salient objects in VFSS images with high performance and resistance to occlusion. Prior to our study, two studies attempted to automate the tracking of hyoid movement by applying deep learning technology [21,27]. We compared the previous studies with our study as follows. Zhang et al. performed bounding box regression from feature maps of various sizes using an SSD network [27]. The study used a larger data set, consisting of 265 subjects, than our data set of 197 subjects. Their model could fully automatically detect the hyoid bone and showed high performance in terms of object size. However, SSD showed a problem of losing balance among the classes in training because objects were not present in all of the bounding boxes with top-down feature extraction only. It also showed relatively unsatisfactory accuracy and had a limitation of tracking failure when passing the mandible. For this reason, the performance of detecting the hyoid bone with SSD using a bounding box was 23.4%∼29% lower than that of other models (Table 2), although it showed relatively less error compared to its low detection performance since SSD uses an anchor box. In another study by Lee et al. [21], a CNN-based online learning algorithm was proposed that can track hyoid motion and predict it even when it passes through the mandible and is blurred. U-Net combined with an attention mechanism was used to detect objects. Their attention U-Net used a featurewise gating mechanism that adjusts the output according to the importance of the features extracted in the top-down approach. However, the attention process was executed in a top-down way similar to the methods of U-Net and SSD. Moreover, there was a possibility that features of different sizes could contribute equally because the weight was not adjusted in combining the features. The performance of detecting the hyoid bone with Attention U-Net using a bounding box was 3.3% higher than that with U-Net, whereas the performance of detecting the coin was 6.3% lower (Table 2). The low performance of detecting the coin with attention U-Net may be due to poor inference performance in the areas that are not visible, originating from the poor performance of bounding box regression. Moreover, their model still requires manual demarcation of a hyoid bone of appropriate size by humans in the first frame. We used both top-down and bottom-up approaches by applying the BiFPN to extract features efficiently and made the features contribute differently according to their size by adjusting the weight in combining the extracted features. Their results are based on a smaller data set, consisting of 77 subjects, than our data set of 197 subjects. In addition, we extracted the global context using the BOT to design a strong network for occlusion and significantly improved the performance in object detection. The proposed BiFPN-U-Net(T) performed well as long as the image quality is secured and showed much higher performance in detecting the hyoid bone and cervical spine than the other models: 2.3∼29% and 1.6∼4.5% higher performance, respectively. The estimated distance of hyoid bone movement by BiFPN-U-Net(T) showed the closest value to the ground truth (Table 3), suggesting the most successful tracking performance compared to the other models. Such good results are probably attributable to the ability of the global context in BOT to extract strong feature information in occlusion cases. Furthermore, the network with BiFPN can detect objects of various sizes more effectively by extracting strong feature information from the size. The results of this study confirmed the higher performance of the BiFPN-U-Net(T) network (mAP = 95.9%, Dice similarity = 90.9%) than the models proposed by Zhang et al. (mAP = 83.1%) [27] and Lee et al. (mAP = 93.1%, Dice similarity = 89.7%) [21]. BiFPN-U-Net(T) also showed the most accurate results in measuring the maximum trajectory distance of the hyoid bone, which is a clinically significant variable.

This study has other considerable advantages, as follows. Since the kinematic properties of the hyoid bone are related to age and risk of penetration–aspiration [11,50] and the viscosities of diet can affect swallowing time [51], we collected a data set aiming to evenly distribute factors such as age, severity of penetration–aspiration, and type of diet. Our data set of 69,389 frames from 197 video files for training the model was annotated manually by three physiatrists skilled in VFSS analysis and contained a 24 mm coin to establish a standard of distance. The point we want to emphasize in our study is that human intervention is not required in the whole process of tracking the hyoid bone in VFSS videos, unlike the previous study in which the salient structures were demarcated manually in the first frames of video images. Nevertheless, the model we first proposed here can track the whole movement and showed better performance in video files with greater lengths compared with the previous models, except for a slightly larger RMSE. The average recording time for each video is 11 s, which is much longer than those of the data sets in other papers (2 to 5 s long in the study of Lee et al. [21], and an average of 1.1 s in the study of Zhang et al. [27]); the data set has 336 frames per video on average, where the shortest video is 2 s long and the longest video is 85 s long. By using longer videos containing movements other than swallowing, such as mastication, we believe that a more robust network could be constructed.

The network proposed in this study showed an RMSE 3.57 mm higher than that of Lee et al., which was 3.44 mm [21]. In addition to using different data sets, the following two factors may have caused the larger error: (1) In the present model, the distance between the hyoid bone and cervical spine was estimated on the basis of the estimated size of the attached coin. The RMSE may be increased by poor estimation of the coin size. (2) The average length of the video files was 11 s, much longer than those in previous studies. This is because our data set videos include not only swallowing but also the oral phase, and the resultant jittering of the head made identification of the hyoid bone more difficult. In this study, we collected 197 VFSS video files from different patients in the same hospital, so the data set may provide limited variability of patient groups. The present model, like previous models, can track only the hyoid bone, and other significant VFSS parameters, such as penetration, aspiration, and time parameters, cannot be measured, which limits the application of the model in real clinical settings.

Other limitations of this study are as follows. The difference between normal and dysphagic conditions was not examined, although the data set consisted of videos taken from individuals with various conditions and characteristics. Further studies are required to determine whether the proposed model is useful in revealing the mechanism of dysphagia and the effect of disease conditions. There are many variables other than the tracking of the hyoid bone for analysis of VFSS. A comprehensive software application that can combine the results of the variables and determine their interactions for use in a clinical setting needs to be developed. A large-scale clinical trial may be necessary to demonstrate its usefulness.

The following abbreviations are used in this manuscript:

## Figures and Tables

**Table 1:** Comparison of the pixel detection performance among models.

	Acc	Recall	Precision	Dice Similarity
U-Net	99.5%	86.9%	92.6%	89.6%
Attention U-Net	99.5%	87.3%	92.3%	89.7%
BiFPN-U-Net(T) (proposed)	99.5%	89.5%	92.4%	90.9%

**Table 2:** Bounding box detection performance comparison among models (IOU = 0.5).

	Hyoid Bone	Cervical Spine	Coin	maP
SSD	62.9%	95.0%	91.3%	83.1%
U-Net	86.3%	97.9%	98.7%	94.3%
Attention U-Net	89.6%	97.5%	92.4%	93.1%
BiFPN-U-Net(T) (proposed)	91.9%	99.5%	96.4%	95.9%

**Table 3:** Estimated distance of hyoid bone movement.

	Horizontal	Vertical	Diagonal	Maximum Diagonal
SSD (1330)	9.92 ± 5.32	9.47 ± 4.6	15.25 ± 6.13	31.38 ± 19.36
U-Net (1342)	8.49 ± 3.91	9.28 ± 4.43	13.85 ± 5.32	29.39 ± 22.4
Attention U-Net (1356)	8.3 ± 2.89	9.51 ± 5.06	14.02 ± 4.9	27.06 ± 14.82
BiFPN-U-Net(T)(proposed) (1360)	8.14 ± 3.32	8.95 ± 3.9	13.28 ± 4.17	22 ± 11.03
Ground Truth (1391)	8.54 ± 3.23	9.66 ± 4.13	14.13 ± 4.18	21.14 ± 6.69

Values are given as mean ± standard deviation (mm).

**Table 4:** Root-mean-square error (RMSE) between the actual distance and the predicted distance for hyoid bone movement (mm).

	Horizontal	Vertical	Diagonal	Maximum Diagonal
SSD	2.22	2.81	4.10	6.20
U-Net	5.02	2.61	6.18	17.05
Attention U-Net	3.89	2.25	5.00	7.46
BiFPN-U-Net(T)(proposed)	2.95	1.37	3.57	3.21

Values are in mm.

## Data Availability

The data presented in this study are available from the corresponding author upon reasonable request.
